# Chronic Immune Activation in Systemic Lupus Erythematosus and the Autoimmune PTPN22 Trp^620^ Risk Allele Drive the Expansion of FOXP3^+^ Regulatory T Cells and PD-1 Expression

**DOI:** 10.3389/fimmu.2019.02606

**Published:** 2019-11-08

**Authors:** Ricardo C. Ferreira, Xaquin Castro Dopico, João J. Oliveira, Daniel B. Rainbow, Jennie H. Yang, Dominik Trzupek, Sarah A. Todd, Mhairi McNeill, Maristella Steri, Valeria Orrù, Edoardo Fiorillo, Daniel J. M. Crouch, Marcin L. Pekalski, Francesco Cucca, Tim I. Tree, Tim J. Vyse, Linda S. Wicker, John A. Todd

**Affiliations:** ^1^JDRF/Wellcome Diabetes and Inflammation Laboratory, Nuffield Department of Medicine, Wellcome Centre for Human Genetics, NIHR Oxford Biomedical Research Centre, University of Oxford, Oxford, United Kingdom; ^2^Cambridge Institute for Medical Research, University of Cambridge, Cambridge, United Kingdom; ^3^Department of Immunobiology, NIHR Biomedical Research Centre, King's College London, London, United Kingdom; ^4^Istituto di Ricerca Genetica e Biomedica, Consiglio Nazionale delle Ricerche (CNR), Rome, Italy; ^5^Dipartimento di Scienze Biomediche, Università degli Studi di Sassari, Sassari, Italy; ^6^Department of Medical & Molecular Genetics, King's College London, Guy's Hospital, London, United Kingdom

**Keywords:** regulatory T cells (Tregs), systemic lupus erythematosus (SLE), autoimmunity, FOXP3, PTPN22 Arg^620^Trp, PD-1, type I interferon, immunotherapy

## Abstract

In systemic lupus erythematosus (SLE), perturbed immunoregulation underpins a pathogenic imbalance between regulatory and effector CD4^+^ T-cell activity. However, to date, the characterization of the CD4^+^ regulatory T cell (Treg) compartment in SLE has yielded conflicting results. Here we show that patients have an increased frequency of CD4^+^FOXP3^+^ cells in circulation owing to a specific expansion of thymically-derived FOXP3^+^HELIOS^+^ Tregs with a demethylated *FOXP3* Treg-specific demethylated region. We found that the Treg expansion was strongly associated with markers of recent immune activation, including PD-1, plasma concentrations of IL-2 and the type I interferon biomarker soluble SIGLEC-1. Since the expression of the negative T-cell signaling molecule PTPN22 is increased and a marker of poor prognosis in SLE, we tested the influence of its missense risk allele Trp^620^ (rs2476601C>T) on Treg frequency. Trp^620^ was reproducibly associated with increased frequencies of thymically-derived Tregs in blood, and increased PD-1 expression on both Tregs and effector T cells (Teffs). Our results support the hypothesis that FOXP3^+^ Tregs are increased in SLE patients as a consequence of a compensatory mechanism in an attempt to regulate pathogenic autoreactive Teff activity. We suggest that restoration of IL-2-mediated homeostatic regulation of FOXP3^+^ Tregs by IL-2 administration could prevent disease flares rather than treating at the height of a disease flare. Moreover, stimulation of PD-1 with specific agonists, perhaps in combination with low-dose IL-2, could be an effective therapeutic strategy in autoimmune disease and in other immune disorders.

## Introduction

Systemic lupus erythematosus (SLE) is an aggressive form of autoimmune disease characterized by a break of self-tolerance and chronic activation of the innate and adaptive immune system. The variety of clinical manifestations associated with SLE patients illustrates the heterogeneity of the condition, and poses a significant challenge to our understanding of its common etiology (1, 2). A defect in IL-2 signaling has been proposed to be involved in the pathogenesis of SLE, disturbing the homeostatic balance between regulatory (Treg) and effector T cell (Teff) function that is essential to maintain self-tolerance (3, 4). Furthermore, genetic evidence from recent genome-wide association studies support a prominent role of the IL-2 signaling pathway in SLE (5). SLE shares a significant overlap with the genetic susceptibility to type 1 diabetes (T1D) (6), another autoimmune disease in which IL-2 signaling defects play a central role in disease etiology. This genetic overlap includes not only the classical HLA class II loci, but also key immune genes, including *PTPN22, IL2-IL21, IL2RA, IL10, IFIH1, TYK2, TNFAIP3, IKZF3, IKZF1*, and *IL2RB-C1QTNF6*, many of which are directly implicated in the IL-2 and type 1 interferon (IFN) pathways and T-cell activation, thereby supporting a common genetically-regulated disease etiology between these two autoimmune conditions.

Tregs characterized by the expression of the transcription factor FOXP3, are critically dependent on IL-2 signaling for survival and function, and have a key role in the maintenance of self-tolerance and tissue integrity (7). Nevertheless, to date, the exact role of FOXP3^+^ Tregs in the etiology of SLE remains elusive, and there are inconsistent reports in the literature regarding the numbers and function of Tregs in this disease (8, 9). One explanation for this is the inconsistent definition of a Treg phenotype, and the appreciation of the increasing level of cellular and functional heterogeneity in this population (10). One example is the identification of a non-conventional subset of Tregs that express low levels of CD25, a key marker along with CD127 that is widely used to define Tregs in flow cytometric analyses, which we (11) and others (12–14) have recently reported to be increased in SLE patients.

Understanding whether there is a genetically-intrinsic Treg defect in SLE patients or whether Treg function is impaired in the sense that a patient's Tregs failed to prevent the disease as a result of disease mechanisms in other cell types, for example Teffs (15), is critical to assess the potential success and development of Treg-targeted therapies in SLE. The PTPN22 gene, encoding the tyrosine phosphatase LYP and shown to act as an inhibitor of T-cell receptor signaling, activation and proliferation, has a common missense variant Arg^620^Trp (rs2476601C>T), the minor allele of which (Trp^620^) predisposes to several autoimmune diseases, including SLE and T1D (16). Transcriptomics analyses of CD8^+^ T cells isolated from SLE patients has revealed a distinct activated T-cell gene expression signature associated with poor clinical prognosis (17). Of note, elevated expression of *PTPN22* was highly correlated with this CD8^+^ T-cell gene expression signature, suggesting that its upregulation could indicate an attempt to regulate Teff hyperactivity during flaring autoimmunity (17). However, to date the exact mechanism by which this missense allele is associated with increased risk of autoimmunity remains uncertain (18), with studies reporting different putative functional effects on multiple cell types, including myeloid cells (19), as well as B and T cells (20, 21).

In the present study, we have performed a detailed flow cytometric characterization of the CD4^+^ FOXP3^+^ Treg compartment in two cohorts of SLE patients, providing a broad cross-sectional representation of the different stages of disease activity. Our results show that thymically-derived FOXP3^+^HELIOS^+^ Tregs, which by definition possess a fully demethylated *FOXP3* Treg-specific demethylated region (TSDR), are expanded in SLE, especially during clinically active disease. Furthermore, Tregs from SLE patients showed an activated phenotype, and their frequency is strongly correlated with the circulating levels of other markers of disease activity and chronic inflammation, including soluble SIGLEC-1 (sSIGLEC-1) and IL-2. We also report a previously uncharacterized association of the PTPN22 Trp^620^ risk allele with increased Treg frequency in blood and with elevated expression of the activation marker PD-1 on both CD45RA^−^ Treg and Teff CD4^+^ T-cell populations.

Taken together, our data support that FOXP3^+^ Treg expansion in SLE is a marker of disease activity, likely as a compensatory mechanism to control excess T-cell activity in the context of a recent autoimmune reaction or flare. These findings are particularly relevant in light of the recent reports of clinical benefit of low-dose IL-2 therapy in active SLE (22–24), and suggest that regulatory functions could be enhanced by restoring the homeostatic balance of IL-2 signaling during the phases of disease remission and delaying or preventing the next flare. Moreover, our data also points to a central role of the PD-1 signaling pathway in the pathogenesis of SLE, and suggests that PD-1 immunomodulation, including PD-1 agonism, could be a therapeutic option to inhibit the proliferation of pathogenic autoreactive Teff cells and selectively restore Treg regulatory homeostasis in SLE.

## Materials and Methods

### Subjects

Discovery cohort (cohort 1) study participants included 34 SLE patients recruited from Guy's and St. Thomas' NHS Foundation Trust. All patients satisfied American College of Rheumatology (ACR) SLE classification criteria and were allocated a disease activity using SLEDAI-2K at the time of sampling. SLE patients from cohort 1 were recruited from a clinic in which the severity of disease was such that none the patients were on high dose oral corticosteroids (>15 mg/day) or B-cell depleting therapy, therefore representing a standard clinical cohort providing a cross-sectional representation of patients with moderate to more severe clinical activity on low-dose immunosuppressive drugs. Healthy volunteers matched for age and sex were recruited from the Cambridge BioResource (CBR). This discovery cohort 1 along with matched controls has been characterized in a previous study, where we originally identified the expansion of a subset of CD25^low^ Tregs in SLE (11).

A replication cohort (cohort 2) of 41 SLE patients and 112 healthy volunteers was recruited from the CBR. The SLE patients from cohort 2 were recruited specifically for this study outside their regular clinic visits, and represent a population-based cohort with good disease management that were otherwise well at the time of bleeding. No additional disease information or ancestry data was available for this cohort of patients.

For the analysis of PTPN22 Arg^620^Trp, 551 subjects of self-reported white ethnicity were selected by genotype from the CBR. A subset of 152 subjects, including 40 homozygotes for the missense PTPN22 Trp^620^ allele, 40 heterozygotes and 73 common Arg^620^ homozygotes were selected to investigate the effect of the PTPN22 Arg^620^Trp variant on T-cell phenotypes.

All adult healthy volunteers reported no family history of autoimmune disease. Baseline characteristics for all participating subjects are summarized in Table 1.

**Table 1:** Baseline characteristics of study participants.

**Cohort**	***N***	**SLEDAI**		**Age (years)**		**Female *N* (%)**
		**Median**	**Range**	**Median**	**Range**	
**Dicovery cohort (cohort 1)**						
SLE	34	4	0–14	36	20–72	32 (94.1%)
Healthy controls	24	–	–	42	22–62	23 (95.8%)
**Replication cohort (cohort 2)**						
SLE	41	–	–	52	21–82	38 (92.7%)
Healthy controls	112	–	–	49	26–78	82 (73.2%)
**PTPN22 genotype-selected cohort (CBR)**						
1/ Whole blood Treg immunophenotyping						
Arg^620^/Arg^620^	362	–	–	47	19–78	239 (66.0%)
Arg^620^/Trp^620^	85	–	–	49	31–77	62 (72.9%)
Trp^620^/Trp^620^	39	–	–	55	35–73	23 (59.0%)
2/ Treg intracellular immunophenotyping						
Arg^620^/Arg^620^	73	–	–	50	26–78	52 (71.2%)
Arg^620^/Trp^620^	39	–	–	49	31–77	30 (76.9%)
Trp^620^/Trp^620^	40	–	–	55	35–73	23 (55.5%)

*Baseline characteristics for the study participants stratified by the study cohorts. SLE, systemic lupus erythematosus; SLEDAI, systemic lupus erythematosus disease activity index; CBR, Genotype selected healthy donors recruited from the Cambridge BioResource*.

### Ethics Statement

All samples and information were collected with written and signed informed consent. Informed consent was obtained from SLE patients (cohort 1—REC Ref: 07/H0718/49). Adult SLE patients (cohort 2) and matching adult healthy volunteers were enrolled from the CBR. The study was approved by the local Peterborough and Fenland research ethics committee (05/Q0106/20 and 08/H0308/153). The study conformed to the Declaration of Helsinki and all local ethical requirements.

### Peripheral Blood Mononuclear Cell Sample Preparation

Peripheral blood mononuclear cells (PBMCs) were isolated by Ficoll gradient centrifugation (Lymphoprep; StemCell Technologies) and cryopreserved in 10% heat-inactivated human AB serum (Sigma), as described previously (25). SLE patients and healthy volunteers were recruited contemporaneously and samples were processed and stored by the same investigators to prevent spurious findings caused by differential sample preparation.

### Flow Cytometry

For intracellular immunostainings, PBMCs were rapidly thawed at 37°C in a water bath, resuspended in X-VIVO 15 (Lonza) +1% heat-inactivated, filtered, human AB serum (Sigma). PBMCs were immunostained with fluorochrome-conjugated antibodies against surface-expressed markers for 30 min at room temperature. Cells were washed and stained for viability discrimination using the Fixable Viability Dye eFluor 780 (eBioscience) for 20 min at 4°C. Fixation and permeabilization was performed using the FOXP3 Fix/Perm Buffer Set (eBioscience) according to the manufacturer's instructions, and cells were then immunostained with fluorochrome-conjugated antibodies against intracellular markers for 45 min at room temperature. To minimize the effect of technical variation of the assay and biological covariates like age and sex, samples were assessed in five batches of 40 on each day over a period of two weeks. Each batch was composed of eight SLE patients and 32 healthy donors (including 16 PTPN22 Arg^620^ common homozygotes, 8 PTPN22 heterozygotes and 8 PTPN22 Trp^620^ homozygotes) matched as closely as possible for age, sex and time of collection. Because we were testing the effect of the rare PTPN22 Trp^620^ non-synonymous allele on Treg frequency, we excluded the rare genotype-selected Trp^620^ homozygous donors from all SLE vs. healthy controls analyses.

For surface immunostainings, 150 μl of whole blood was incubated with fluorochrome-conjugated antibodies at room temperature for 45 min, within 4 h of phlebotomy. Red cells were then lysed (BD FACS Lysing Solution) and washed prior to sample acquisition.

Information on all fluorochrome-conjugated antibodies and immunostaining panels used in this study is provided in Supplementary Table 1. All experiments were performed in an anonymized, blinded manner without prior knowledge of disease state. Immunostained samples were acquired using a BD Fortessa (BD Biosciences) flow cytometer with FACSDiva software (BD Biosciences) and analyzed using FlowJo (Tree Star, Inc.). Dead cells were excluded based on the eFluor780 Fixable Viability Dye. Samples from cohorts 1 and 2 were processed and acquired on the flow cytometer within a period of 11 months of each other.

### Analysis of the *FOXP3* TSDR Demethylation

Three SLE patients from cohort 2 (enrolled from CBR) were recalled based on the presence of a type 1 IFN signature and high frequency of CD25^low^ FOXP3^+^ T cells. Total CD4^+^ T cells were enriched from 50 ml whole blood using RosetteSep (StemCell Technologies) and immunostained with fluorochrome-conjugated antibodies as described above (see Supplementary Table 1). Memory CD45RA^−^ FOXP3^+^HELIOS^+^ Tregs from the (i) CD127^low^CD25^low^ and (ii) CD127^low^CD25^high^ subpopulations and a negative control subset of CD45RA^−^ CD127^+^CD25^int/low^ Teffs were FACS sorted using a BD Aria Fusion flow cytometer (BD Biosciences). Methylation of the *FOXP3* TSDR was performed using a next-generation sequencing method, as described previously (26).

### Plasma Soluble SIGLEC-1 and IL-2 Measurements

Soluble SIGLEC-1 (sSIGLEC-1) concentrations were measured in plasma samples from all study participants using a time-resolved fluorescence immunoassay, as described previously (27).

Circulating IL-2 concentrations were measured in plasma samples from the 41 SLE patients from cohort 2 by Single-Molecule Array (SIMOA) digital ELISA (Quanterix) according to the manufacturer's instructions. Measurements were performed in plasma samples that had only undergone one freeze-thaw cycle.

### *In vitro* Treg Suppression and Teff Proliferation Assays

Total Tregs (CD4^+^ CD127^low^CD25^hi^) and autologous memory Teffs (mTeffs; CD4^+^CD45RA^−^ CD127^+^CD25^int−low^) or naïve Teffs (nTeffs; CD4^+^CD45RA^+^ CD127^+^CD25^int−low^) were FACS sorted from cryopreserved PBMCs from a subset of 27 common Arg^620^ homozygous and 24 age- and sex-matched rare Trp^620^ homozygous donors into 96 well plates using a BD FACS Aria II. Samples were stimulated with anti-CD2/3/28 beads (Miltenyi Biotec) and incubated at 37°C, 5% CO_2_ for 6 days. Proliferation was assessed by the addition of 0.5 μCi/well [^3^H]thymidine (PerkinElmer, Waltham, MA) for the final 18 h co-culture. All conditions were run in quintuplicate and proliferation readings ([^3^H]thymidine incorporation rate—CPM) averaged. Tregs were stimulated in the absence of mTeffs in triplicate, and no proliferation above background was observed in any of the donors. The percentage suppression in each culture was calculated using the following formula: % suppression = 100 – [(CPM in the presence of Tregs ÷ CPM in the absence of Tregs) × 100].

### Transcriptional Profiling of Total CD4^+^ T Cells

Gene expression profiling was performed by NanoString, using the pre-designed nCounter Human Immunology v2 Panel (NanoString Technologies). Total CD4^+^ T cells from a subset of (i) four SLE patients with previously identified high transcriptional IFN signature, (ii) two SLE patients with low IFN signature, and (iii) four healthy controls, were obtained by negative selection (StemCell Technologies) from cryopreserved PBMCs. A total of 25,000 cells were collected into RLT lysis buffer (Qiagen) following *in vitro* stimulation for 120 min with PMA and ionomycin cocktail without addition of protein transport inhibitors (eBiosciences). RNA was extracted using the RNAeasy Micro Plus kit (Qiagen), with gDNA cleanup, and hybridized to the NanoString CodeSets, following manufacturer's instructions. Expression levels were assessed using an nCounter Sprint instrument (NanoString Technologies). Data were processed using the nSolver Analysis Software following normalization of the raw read counts to the geometric mean of positive control spike-ins, and the gene expression of 15 selected housekeeping genes that were found to have low variability following *in vitro* stimulation, as previously described (28).

### Targeted Single-Cell RNA-Sequencing Analyses

Single-cell RNA sequencing data, was obtained from one SLE patient with an expanded CD25^low^FOXP3^+^ Treg population, one type 1 diabetes (T1D) patient and one healthy control (HC), presented in Trzupek et al. (29). Briefly, simultaneous mRNA expression of 397 genes (Human Response Panel; BD Biosciences) and protein expression of 24 T-cell expressed targets (see Supplementary Table 1), were assessed at the single-cell level using the BD Rhapsody and AbSeq system (BD Biosciences), as previously described (29). Flow sorted CD4^+^ T-cell subsets from each donor were barcoded using oligo-conjugated antibodies (single-cell multiplexing kit; BD Biosciences). For this study, we extracted and reanalyzed the data from sorted CD127^low^CD25^low^ (*N* = 7,115) and CD127^low^CD25^hi^ (*N* = 7,711) T cells passing QC. Detailed description of sorting strategy, library preparation and sequencing is presented in Trzupek et al. (29). Data analysis and QC was performed following the BD Biosciences Rhapsody pipeline (BD Biosciences) and the R package Seurat 3.0 (30). Uniform Manifold Approximation and Projection (UMAP) was used for dimensionality reduction. Differential expression analysis was performed using a tailored hurdle model from MAST package (31) used by Seurat.

### Genotyping and Quality Control

rs2476601 genotypes for the 551 PBMC bank subjects were obtained using the Illumina ImmunoChip. Genotyping of 196,524 SNPs was performed in two batches of 191 and 360 samples. 59,556 SNPs were excluded from the analysis because they were either monomorphic in at least one of the genotyping batches (*n* = 40,091) or corresponded to A/T or G/C polymorphisms (*n* = 19,465), for which the correct genotype assignment could not be unambiguously determined. SNPs were tested for departure from Hardy-Weinberg equilibrium within either batch or with batches combined, and 2,183 with significant departures at *P* < 10^−5^ were discarded. Batches were tested against each other for genotypic differences using Fisher's Exact Test (3 × 2 genotypic test) and 674 SNPs with significant genotype differences at *P* < 10^−5^ were discarded. rs2476601 was not subjected to these quality control (QC) steps as subjects had been selected for inclusion in the PBMC bank based using their genotypes at this position. After removal of 10,073 SNPs with missingness > 1%, the remaining 124,038 SNPs were used to apply subject-level QC filters. The 3 subjects found to have missing genotypes at > 1% of SNPs were discarded, in addition to 15 that were Principal Component (PC) outliers, leaving 533 subjects. Outliers were defined as individuals with >6 standard deviations from the mean for any of the top 10 PCs, as computed from the post-QC SNP data (using 93,546 SNPs with MAF > 5%). After outlier removal, PCs were recomputed and the process iterated until no outliers remained. QC procedures were performed in PLINK (32) and R.

### Statistical Analyses

Statistical analyses were performed using Prism software (GraphPad), PLINK and R. Frequency of the interrogated immune subsets were compared between SLE patients and healthy donors or PTPN22 genotype groups using two-tailed Student's *t*-tests. The correlations between immune parameters and the plasma concentrations of sSIGLEC-1 and IL-2 were calculated using linear regression analysis. Given that plasma protein concentrations showed moderate to strong right skew that violated the assumption of normality, the phenotypes were log-transformed before statistical testing. The effects of age, sex and time of collection were controlled by the experimental design used in this study and, therefore, not included as additional covariates.

The relationship between Treg frequency in blood and rs2476601 genotype was analyzed by regressing the adjusted Treg frequency measurement against the number of minor alleles. This was performed using 486 individuals who had both Treg frequency and genotype data post-QC. For subjects who had multiple Treg frequency measurements (*n* = 20), the mean was used. To account for a potential systematic variation in the estimation of the Treg frequencies by flow cytometry during the duration of the study, Treg frequencies were corrected for number of days since the first measurement and for batch (there were two batches of 383 and 120 individuals), by linearly regressing out these variables. Linear regression was used to estimate the effect of each missense PTPN22 Trp^620^ allele (rs2476601) with Treg frequency, for the 486 individuals with both Treg frequency and rs2476601 genotypes available. Effects of genetic ancestry and relatedness between subjects were corrected for via the inclusion of 10 PCs computed using the 124,038 SNPs and 533 subjects with genotypes remaining after QC. A similar analysis was done to assess the relationship between the frequency of the CD45RA^+^ naïve and CD45RA^−^ memory Tregs and rs2476601 genotypes (*n* = 485; one donor was excluded from this analysis for missing CD45RA flow cytometric data). Age was strongly correlated with these phenotypes, so was regressed out in addition to batch and number of days since the start of the experiment.

Replication was performed using 3,437 Sardinian PBMC samples and the combined 3,923 individuals were meta-analyzed using Fisher's method for combining *P*-values and inverse-variance weighting of parameter estimates. Summary statistics were scaled by the Treg standard deviation prior to meta-analysis before rescaling final estimates via the estimate from the UK group (SD = 1.77).

## Results

### Frequency of CD4^+^ FOXP3^+^ Tregs Is Increased in Peripheral Blood From SLE Patients

**Figure 1:**
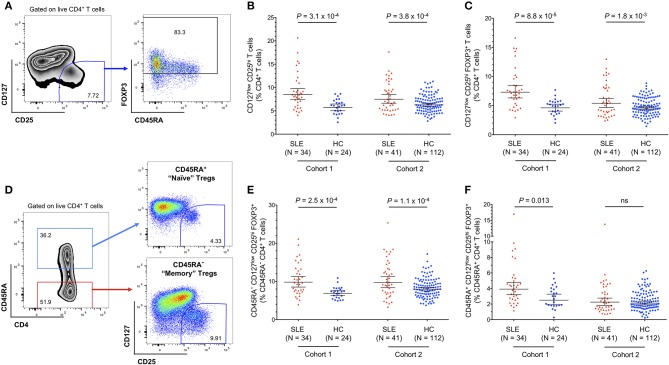
Regulatory CD4^+^ T cell (Treg) frequency is increased in the circulation of SLE patients. **(A)** Gating strategy for the delineation of CD4^+^ regulatory T cells (Tregs). **(B,C)** Scatter plots depict the frequency (geometric mean ± 95% CI) of CD127^low^CD25^hi^ Tregs **(B)**, and CD127^low^CD25^hi^ FOXP3^+^ Tregs **(C)** in PBMCs isolated from SLE patients (depicted in red) and matching healthy volunteers (depicted in blue). **(D)** Gating strategy for the delineation of the CD45RA^+^ naïve and CD45RA^−^ memory CD4^+^ T-cell compartments. **(E,F)** Scatter plots depict the frequency (geometric mean ± 95% CI) of memory CD45RA^−^ **(E)**, and naïve CD45RA^+^ **(F)** CD127^low^CD25^hi^ FOXP3^+^ Tregs. Data were obtained from a discovery clinic-attending cohort (cohort 1) consisting of 34 SLE patients and 24 healthy volunteers, and from a population-based replication cohort (cohort 2) consisting of 41 SLE patients and 112 healthy volunteers. *P*-values were calculated using two-tailed Student's *t*-tests comparing the frequency of the assessed immune subsets in patients and controls. SLE, systemic lupus erythematosus; HC, healthy control; ns, not significant.

To investigate the circulating CD4^+^ Treg compartment of SLE patients, we performed a detailed flow cytometric characterization of cryopreserved PBMCs from two cohorts of patients: (i) a clinic-attending—cohort 1- of patients recruited at the time of their regular clinic visit; and (ii) a population-based cohort—cohort 2—of patients recruited outside of their regular clinic visits through the Cambridge BioResource. To avoid potential discrepancies caused by how the Treg compartment was defined, in this study, we have delineated Tregs both through their surface expression of IL-7R/CD127 and IL-2RA/CD25 (CD127^low^CD25^hi^ Tregs), or by further restricting to the cells that express the canonical Treg transcription factor FOXP3 (FOXP3^+^ CD127^low^CD25^hi^ Tregs; Figure 1A). Regardless of how Tregs were delineated, we found a consistent increase in the frequency of both CD127^low^CD25^hi^ (5.7 vs. 8.5% and 6.3 vs. 7.5% in cohorts 1 and 2, respectively; Figure 1B) and FOXP3^+^ CD127^low^CD25^hi^ (4.6 vs. 7.3% and 4.6 vs. 5.4% in cohorts 1 and 2, respectively; Figure 1C) Tregs in blood from SLE patients compared to age-matched healthy volunteers. The increased frequency of Tregs was more pronounced in patients from cohort 1, likely reflecting the increased level of disease activity in that cohort of patients. In addition, the expansion of the FOXP3^+^ Tregs in SLE patients was higher in the memory CD45RA^−^ compartment (Figures 1D,E) compared to the naïve CD45RA^+^ compartment (Figures 1D,F), suggesting that the observed increased Treg frequency is not a direct effect of an increased thymic Treg output in SLE patients, but rather is a regulatory response to the inflammation caused by activated immune cells mediating the autoimmune disease. In particular, increased IL-2 production by autoantigen-specific Teffs could promote the expansion of Tregs activated in the local, inflammatory environment.

**Figure 2:**
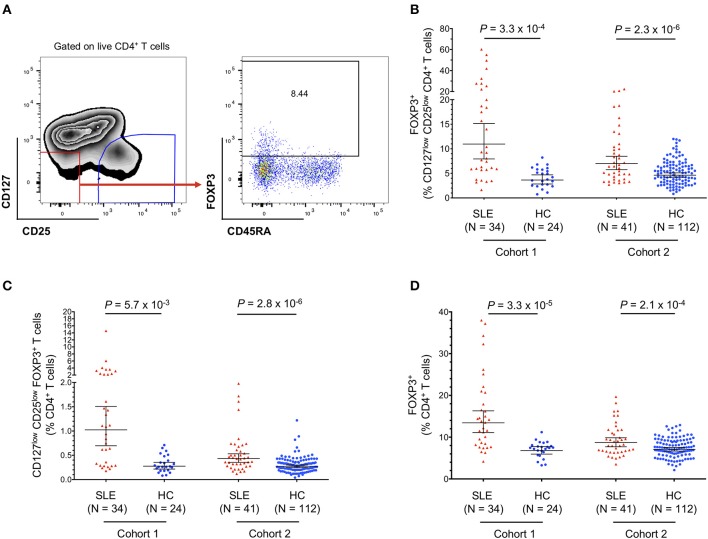
Frequency of FOXP3^+^ T cells in SLE patients is increased in both CD25^low^ and CD25^hi^ CD4^+^ T-cell subsets. **(A)** gating strategy for the delineation of the CD25^low^FOXP3^+^ Treg subset. **(B,C)** Scatter plots depict the frequency (geometric mean ± 95% CI) of CD25^low^FOXP3^+^ Tregs in SLE patients (red) and matching healthy volunteers (blue), defined as the frequency of FOXP3^+^ cells among CD127^low^CD25^low^ T cells **(B)** or as the frequency of CD127^low^CD25^low^FOXP3^+^ T cells among total CD4^+^ T cells **(C)**. **(D)** Scatter plot depicts the frequency (geometric mean ± 95% CI) of total CD4^+^FOXP3^+^ Tregs in SLE patients (red) and matching healthy volunteers (blue), defined as the frequency of FOXP3^+^ cells among total CD4^+^ T cells. Data were obtained from a discovery clinic-attending cohort (cohort 1) consisting of 34 SLE patients and 24 healthy volunteers, and from a population-based replication cohort (cohort 2) consisting of 41 SLE patients and 112 healthy volunteers. *P*-values were calculated using two-tailed Student's *t*-tests comparing the frequency of the assessed immune subsets in patients and controls. SLE, systemic lupus erythematosus; HC, healthy control.

Consistent with our previous data (11), we confirmed that the expansion of FOXP3^+^ cells was also observed within the CD127^low^CD25^low^ compartment (Figures 2A,B). Frequencies of CD25^low^FOXP3^+^ among total CD4^+^ T cells are usually very low in healthy donors (0.04–1.22%; Figure 2C), but were found to be substantially increased among SLE patients, with frequencies as high as 14.6% of total CD4^+^ T cells (Figure 2C). We also noted that the frequency of CD25^low^FOXP3^+^ Tregs displayed a bimodal distribution among SLE patients, which was particularly noteworthy within the clinic-attending patients from cohort 1 (Figure 2C). In agreement with the observed expansion of FOXP3^+^ T cells in SLE patients in both the conventional CD25^hi^ and the non-conventional CD25^low^ subpopulations, we observed a significant increase in the frequency of total CD4^+^ FOXP3^+^ cells in SLE patients (Figure 2D).

### Expanded Tregs in SLE Patients Are Predominantly FOXP3^+^HELIOS^+^ and Are Demethylated at the FOXP3 TSDR

**Figure 3:**
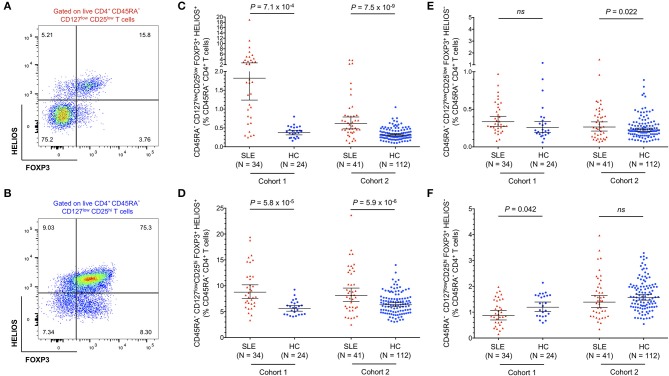
Expanded FOXP3^+^ Tregs in SLE patients are predominantly HELIOS^+^. **(A,B)** Gating strategy for the delineation of the CD45RA^−^ CD25^low^ **(A)** and CD25^hi^ **(B)** Treg subsets stratified by the expression of the canonical Treg transcription factors FOXP3 and HELIOS. **(C,D)** Scatter plots depict the frequency (geometric mean ± 95% CI) of CD25^low^ FOXP3^+^HELIOS^+^ **(C)**, and CD25^hi^ FOXP3^+^HELIOS^+^ **(D)** Tregs in SLE patients (red) and matched healthy volunteers (blue). **(E,F)** Scatter plots depict the frequency (geometric mean ± 95% CI) of CD25^low^ FOXP3^+^HELIOS^−^ **(E)**, and CD25^hi^ FOXP3^+^HELIOS^−^ **(F)** Tregs in SLE patients and matched healthy volunteers. Data were obtained from a discovery clinic-attending cohort (cohort 1) consisting of 34 SLE patients and 24 healthy volunteers, and from a population-based replication cohort (cohort 2) consisting of 41 SLE patients and 112 healthy volunteers. *P*-values were calculated using two-tailed Student's *t*-tests comparing the frequency of the assessed immune subsets in patients and controls. SLE, systemic lupus erythematosus; HC, healthy control; ns, not significant.

To further investigate the origin of the expanded CD45RA^−^ FOXP3^+^ Tregs in SLE patients we further stratified the distribution of both CD25^low^ (Figure 3A) and CD25^hi^ (Figure 3B) Treg populations according to the expression of HELIOS, a key transcription factor required to maintain the Treg transcriptional programme and suppressive function (33). In both subsets, we found a higher frequency of FOXP3^+^HELIOS^+^ Tregs in blood from SLE patients compared to matched healthy volunteers (Figures 3C,D). We have shown previously that essentially all FOXP3^+^HELIOS^+^ Tregs are demethylated at the *FOXP3* TSDR (11) In contrast, we found no evidence for an increase in the frequency of FOXP3^+^HELIOS^−^ Tregs, a subset in which only a portion of the cells have a demethylated *FOXP3* TSDR (11) and that we have previously shown to be enriched in cells with the capacity of producing pro-inflammatory cytokines (28), in either the CD25^low^ (Figure 3E) or CD25^hi^ (Figure 3F) compartments. These data indicate that the increased Treg frequency observed in SLE patients results from the specific expansion of thymically-derived FOXP3^+^ Tregs.

To further confirm this hypothesis, we recalled three SLE patients from cohort 2 that showed an expanded FOXP3^+^ Treg compartment, and assessed the epigenetic profile at the *FOXP3* locus in freshly isolated CD25^low^ and CD25^hi^ FOXP3^+^HELIOS^+^ Tregs (Supplementary Figure 1A). In sharp contrast with CD25^−^CD45RA^−^ memory Teff cells, we found that both CD25^low^ and CD25^hi^ CD45RA^−^FOXP3^+^HELIOS^+^ Tregs displayed a fully demethylated *FOXP3* TSDR (Supplementary Figure 1B), consistent with a stable constitutive expression of *FOXP3* and therefore a thymic origin and Treg identity.

### Single-Cell RNA-Sequencing Reveals Phenotypic Similarities Between *ex vivo* Isolated CD25^low^FOXP3^+^ T Cells and Conventional CD25^hi^FOXP3^+^ Tregs

**Figure 4:**
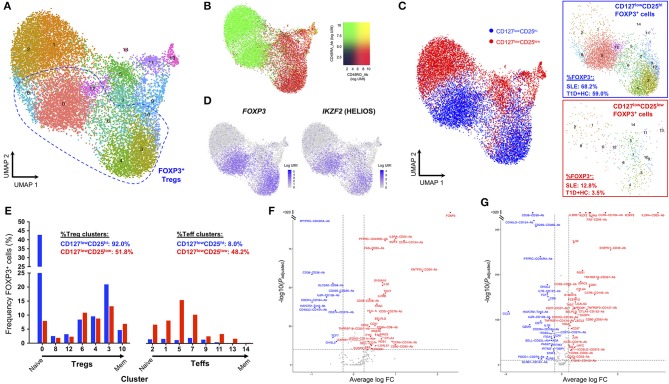
Single-cell RNA-sequencing reveals phenotypic similarities between CD25^low^FOXP3^+^ T cells and conventional CD25^hi^FOXP3^+^ Tregs. **(A)** Uniform manifold approximation projection (UMAP) plot depicting clustering of all captured CD4^+^ single cells using the combined proteomics and transcriptomics data. Data were extracted from a targeted single-cell RNA-sequencing dataset (ref. 29), and depicts the analysis of pre-sorted CD127^low^CD25^hi^ (*N* = 7,711) and CD127^low^CD25^low^ (*N* = 7,115) T cells from one SLE patient and two control donors, including one type 1 diabetes (T1D) patient and one healthy donor. **(B)** Expression levels of the CD45RA (black to green) and CD45RO (black to red) isoforms using oligo-conjugated antibodies. **(C)** Expression of the canonical Treg transcription factors FOXP3 and HELIOS in the identified resting CD4^+^ T-cell clusters. **(D)** Detection of FOXP3^+^ cells (as assessed by the expression of >=1 copy of *FOXP3* in each single cell) in either the pre-sorted CD127^low^CD25^hi^ (blue) or CD127^low^CD25^low^ (red) populations. Relative frequencies of FOXP3^+^ cells in SLE and control donors is indicated in the figure. **(E)** Frequency of FOXP3^+^ cells from either the CD127^low^CD25^hi^ or CD127^low^CD25^low^ populations in each identified regulatory T cell (Treg) or effector T cell (Teff) cluster. Clusters were ordered according to their relative positioning along the naïve to memory (Mem) differentiation axis. **(F,G)** Volcano plots depict the differential expression of the assessed genes at the mRNA (*N* = 397 genes) and protein (*N* = 24) level between: (i) CD127^low^CD25^low^ FOXP3^+^ vs. CD127^low^CD25^low^ FOXP3^−^ T cells **(F)**; and (ii) CD127^low^CD25^hi^ FOXP3^+^ Tregs vs. CD127^low^CD25^low^ FOXP3^−^ T cells **(G)**.

Currently there are no identified surface markers that allow the isolation of the identified CD25^low^FOXP3^+^ T cells, which precludes the unequivocal characterization of their putative suppressive capacity. This is particularly relevant in SLE patients with active disease, where the disease-specific environment could affect cell function. Therefore, to further investigate the nature of CD25^low^FOXP3^+^ T cells, we employed a recently developed targeted single-cell RNA-sequencing approach combining mRNA and protein quantification (29), to characterize the FOXP3^+^ cells within both CD127^low^CD25^low^ and CD127^low^CD25^hi^ populations from one SLE patient displaying an expanded Treg compartment, as well as two control donors (one type 1 diabetic and one healthy donor). Clustering analysis of the combined mRNA and protein expression from all *ex vivo* isolated CD127^low^CD25^low^ (*n* = 7,115) and CD127^low^CD25^hi^ (*n* = 7,711) cells, revealed diverse functional T-cell subsets, clustering along a naïve-memory differentiation axis, including distinct Treg clusters marked by the expression of *FOXP3* and *IKZF2* (encoding HELIOS; Figures 4A–C and Supplementary Figure 2A). To identify FOXP3^+^ cells within the two assessed T-cell populations, we then isolated cells with detectable FOXP3 expression. We noted that in the SLE patient 68.2% of CD127^low^CD25^hi^ and 12.8% CD127^low^CD25^low^ T cells expressed FOXP3 at the mRNA level (Figure 4D). This compared to 83.8 and 20.8%, respectively for the expression of FOXP3 detected at the protein level by flow cytometry in the same patient, and is consistent with the lower sensitivity of mRNA detection, especially of lowly expressed genes, by scRNA-seq methods. Importantly, CD25^low^FOXP3^+^ cells clustered together with the conventional CD25^hi^FOXP3^+^ Tregs, with similar distribution across the different identified Treg subsets (Figure 4E). The only notable exception was a marked absence of CD45RA^+^ naïve Tregs among CD25^low^FOXP3^+^ cells, which is consistent with the activated phenotype previously observed among this subset (11). Furthermore, differential expression analysis revealed a distinct upregulation of classical Treg signature genes, including IL-2RA and HELIOS, within CD25^low^FOXP3^+^ as compared to their respective CD25^low^FOXP3^−^ counterparts (Figure 4F). A very similar Treg signature was observed when comparing conventional CD25^hi^FOXP3^+^ Tregs with the same CD25^low^FOXP3^−^ Teff cell population (Figure 4G), which further supports the Treg function and identity of the majority of *ex vivo* isolated CD25^low^FOXP3^+^ cells.

Nevertheless, we note that ~48.2% of CD25^low^FOXP3^+^ cells clustered within non-Treg subsets, compared to only 8% within conventional CD25^hi^FOXP3^+^ Tregs (Figure 4E). These results demonstrate the increased heterogeneity of the CD127^low^CD25^low^ population and the need to identify further surface markers to isolate CD25^low^FOXP3^+^ cells in order to assess their function. Furthermore, these data also indicate that a proportion of CD25^low^FOXP3^+^ cells could represent either non-conventional Treg subsets or activated Teffs, transiently upregulating FOXP3 at the protein level. Of note, the relative distribution of either CD25^hi^FOXP3^+^ CD25^low^FOXP3^+^ cells within the different T-cell clusters was similar between the SLE and the two control donors (Supplementary Figures 2B,C), which suggests there is no evidence for a disease-specific enrichment in putative activated CD25^low^FOXP3^+^ Teff subsets.

### Memory CD25^hi^FOXP3^+^HELIOS^+^ Tregs From SLE Patients Display an Activated Phenotype

**Figure 5:**
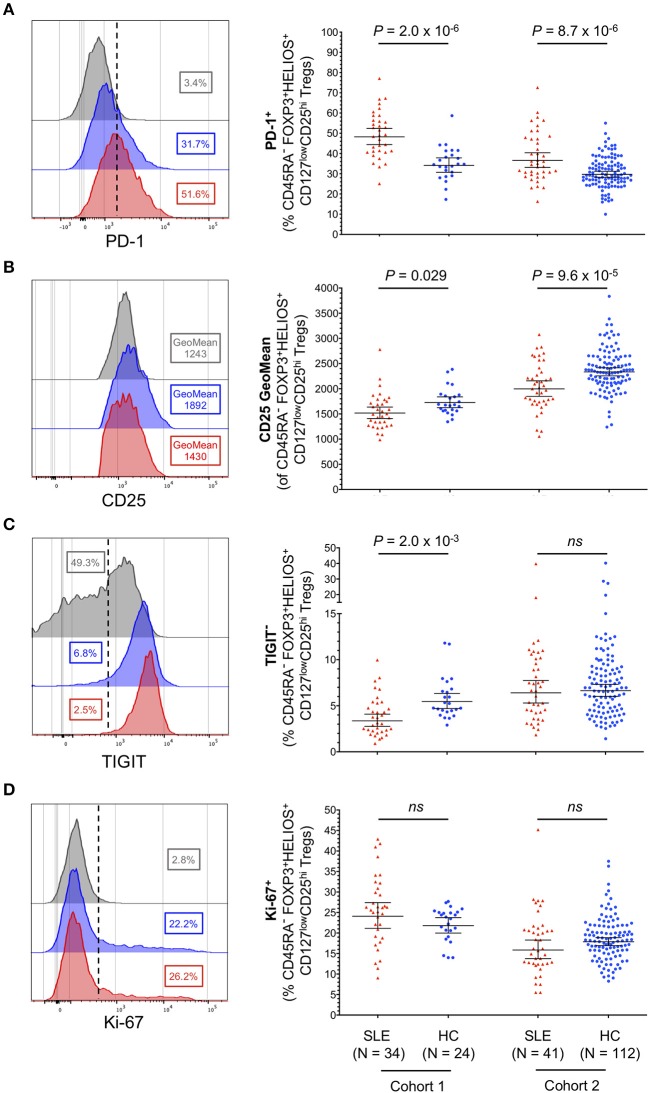
Memory FOXP3^+^HELIOS^+^ Tregs from SLE patients display hallmarks of recent immune activation. **(A–D)** Representative histograms and summary scatter plots depict the frequency (geometric mean ± 95% CI) of: (i) PD-1^+^ **(A)**; (ii) CD25 mean fluorescence intensity **(B)**; (iii) TIGIT^−^ **(C)**; and (iv) Ki-67^+^ **(D)** cells within CD45RA^−^ CD25^hi^ FOXP3^+^HELIOS^+^ Tregs. Data were obtained from a discovery clinic-attending cohort (cohort 1) consisting of 34 SLE patients (depicted in red) and 24 healthy volunteers (depicted in blue) and from a population-based replication cohort (cohort 2) consisting of 41 SLE patients and 112 healthy volunteers. Samples from cohorts 1 and 2 were processed within a period of 11 months of each other. For each parameter an illustrative histogram is provided as a reference depicting the expression of the marker in the CD45RA^+^ FOXP3^+^HELIOS^+^ naïve Treg population (depicted in gray). *P*-values were calculated using two-tailed Student's *t*-tests comparing the frequency of the assessed immune subsets in patients and controls. SLE, systemic lupus erythematosus; HC, healthy control; ns, not significant.

Having established that CD45RA^−^ CD25^hi^FOXP3^+^HELIOS^+^ Tregs represented the majority of expanded Tregs in SLE patients, we next performed a detailed phenotypic characterization of this subset to gain further insight into their potential function and causes of expansion. The main distinguishing features of CD25^hi^FOXP3^+^HELIOS^+^ Tregs isolated from SLE patients were the increased expression of the activation marker PD-1 (Figure 5A) and the reduced expression of CD25 on a per cell basis (Figure 5B). The phenotypic changes associated with CD25^hi^ FOXP3^+^HELIOS^+^ memory Tregs suggest that these cells have expanded in SLE patients in response to an inflammatory, autoimmune reaction; the chronic stimulation could cause CD25 levels on Tregs to decrease due to IL-2RA mRNA down-regulation or to cleavage of the CD25 molecule from the cell surface. Such mechanisms to decrease CD25 levels could account for the increased frequency of CD45RA^−^ CD25^low^FOXP3^+^HELIOS^+^ Tregs in SLE patients. Consistent with this hypothesis, we found a very strong correlation between the frequency of PD-1^+^ cells in CD45RA^−^ CD25^hi^FOXP3^+^HELIOS^+^ Tregs and CD45RA^−^ Teffs in both SLE patients and healthy donors (Supplementary Figures 3A,B). suggesting that expression of PD-1 is a sensitive marker of immune activation on both populations and that as more Teffs are activated there is a compensatory, homeostatic, increase in Treg activation. We noted that expression levels of PD-1 on CD45RA^−^ Teffs are higher than their CD45RA^−^ FOXP3^+^HELIOS^+^ Treg counterparts in both patients and healthy donors, and similar MFI values were observed for the two subsets between patients and controls (Supplementary Figures 3C,D).

Conversely, we found no increase in the frequency of TIGIT^−^ Tregs (Figure 5C), a subset of cells that we have previously shown to contain cytokine-producing T cells and to be enriched for a subset of Th17-like Tregs (28). Furthermore, we did not find significant changes associated with the expression of conventional functional Treg markers, CD15s, CD226, CD161, and HLA-DR (Supplementary Figure 4) or in the expression of the proliferation marker Ki-67 (Figure 5D) in CD45RA^−^ CD25^hi^FOXP3^+^HELIOS^+^ Tregs from SLE patients. Together, these data reveal that the increased frequency of FOXP3^+^ Tregs observed in SLE patients is not caused by a selective expansion of non-conventional or peripherally induced Tregs, which could be less functional or even promoting the production of pro-inflammatory cytokines, but rather by the expansion of bona-fide thymically-derived FOXP3^+^HELIOS^+^ Tregs, showing the hallmarks of suppressive functions.

Similarly to the phenotype of CD45RA^−^ FOXP3^+^HELIOS^+^ Tregs, CD45RA^−^ Teffs from SLE patients also showed evidence for increased expression of T-cell activation and proliferation markers, namely PD-1, Ki-67, TIGIT and CD15s (Supplementary Figure 5). The increased expression of these markers was only significantly increased in the clinic-attending cohort 1, and therefore could reflect aggressive autoreactive Teff cell and chronic activation in patients with active disease flares.

### Frequency of FOXP3^+^ Tregs in SLE Patients Is Highly Correlated With Increased Circulating Levels of Type I Interferon and IL-2

**Figure 6:**
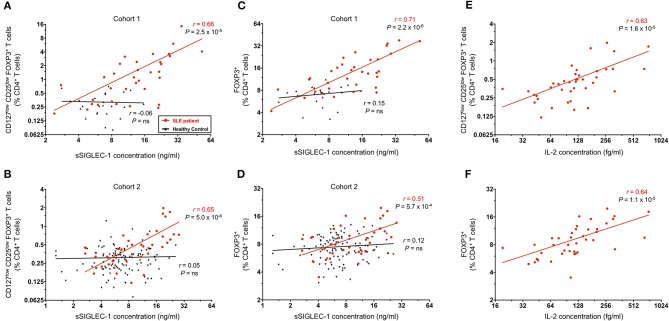
FOXP3^+^ Treg expansion is a marker of recent autoimmune reaction. **(A,B)** Correlation between the frequency of CD25^low^FOXP3^+^ Tregs in blood and the circulating concentration of the plasma type I interferon (IFN) marker soluble SIGLEC-1 (sSIGLEC-1). Data shown depict the correlation in SLE patients (red) and matched healthy volunteers (black) in either the discovery clinic-attending cohort—cohort 1 **(A)** or the replication population-based cohort—cohort 2 **(B)**. **(C,D)** Data shown depict the correlation between the total CD4^+^FOXP3^+^ CD45RA^−^ memory Tregs and the plasma concentration of sSIGLEC-1 in cohort 1 **(C)** and cohort 2 **(D)**. **(E,F)** Data shown depict the correlation between the frequency of CD25^low^FOXP3^+^ **(E)** or total FOXP3^+^ **(F)** CD45RA^−^ memory Tregs and the plasma concentration of IL-2 measured in the 41 SLE patients from cohort 2. The correlation coefficients (r) and the respective *P*-values from the linear regression in SLE patients and healthy controls are shown in the plots.

Recently, we have reported that the plasma concentration of the soluble form of the adhesion receptor SIGLEC-1 (sSIGLEC-1) is sensitive marker of the type I IFN transcriptional signature (27). In addition, we have shown that the concentration of sSIGLEC-1 was also associated with increased disease activity in SLE patients. In the present study, we have found a very strong correlation between the concentration of sSIGLEC-1 in plasma and the frequency of CD25^low^FOXP3^+^ Tregs in SLE patients, but not in healthy controls, in both the clinic-attending (*r* = 0.66, *P* = 2.5 × 10^−5^; Figure 6A) and in the population-based (*r* = 0.65, *P* = 5.0 × 10^−6^; Figure 6B) cohorts. This association was not restricted to the CD25^low^FOXP3^+^ subset, but also observed with total CD4^+^ FOXP3^+^ Tregs (Figures 6C,D), particularly in the clinical cohort (*r* = 0.71, *P* = 2.2 × 10^−6^; Figure 6C). These findings demonstrate that the expansion of FOXP3^+^ Tregs in SLE is correlated with increased type I IFN signaling, which is known to play a major etiological role in the progression of the disease.

To investigate if the observed Treg expansion could also be recapitulated at the transcriptional level, we next investigated the transcriptional profile of total CD4^+^ T cells in a subset of (i) four SLE patients where we had previously detected a high transcriptional IFN signature (IFN^hi^) in total PBMCs (27); (ii) two SLE patients with low IFN signature (IFN^low^); and (iii) four healthy donors (Supplementary Figure 6A and Supplementary Table 2). Although it is well-established that myeloid cells are the main contributors to the transcriptional whole blood IFN signature observed in SLE patients, we observed that the expression of two canonical IFN signature genes (*MX1* and *IFH1*), but not a housekeeping gene (*HPRT1*) were highly correlated with the quantitative IFN signature score (Supplementary Figure 6B), indicating that total CD4^+^ T cells are also contributing to this peripheral transcriptional signature. Furthermore, in agreement with the observed FOXP3^+^ Treg expansion in patients with increased type I IFN signaling, we were able to detect increased expression of the classical Treg signature genes *FOXP3* and *IKZF2* (encoding HELIOS) in IFN^hi^ patients. In contrast, mRNA expression of other markers of activated Tregs, such as *PDCD1* (encoding PD-1), *IL-2RA*, and *HLA-DRA*, were not associated, which is consistent with their more heterogeneous expression pattern in other activated CD4^+^ Teff subsets (Supplementary Figures 6C–E). Further supporting the correlation between protein and mRNA levels, we detected a remarkable correlation between the expression of *FOXP3* and *IKZF2* with the frequency of FOXP3^+^ cells in both total CD4^+^ or CD127^low^CD25^low^ T cells (Supplementary Figures 6F,G), demonstrating that the observed Treg expansion in SLE patients can also be detected through the transcription of classical Treg signature genes in circulating CD4^+^ T cells.

In addition to measuring the levels of type I IFN in circulation, we have also employed a sensitive single molecule digital ELISA assay to measure the circulating concentrations of IL-2 in plasma samples from all 42 SLE patients in the population-based cohort 2. We found a strong correlation between the plasma IL-2 concentration and frequency of both CD25^low^FOXP3^+^ (*r* = 0.63, *P* = 1.6 × 10^−5^; Figure 6E) and total CD4^+^ FOXP3^+^ Tregs (*r* = 0.64, *P* = 1.1 × 10^−6^; Figure 6F). Although we could not attempt to replicate these findings in the clinic-attending cohort of patients due to insufficient plasma volume, it is noteworthy that such a strong correlation could be observed even in samples with relatively modest concentrations of IL-2, which our data suggest could be even higher during flares of the disease.

### The PTPN22 Autoimmune Risk Allele Trp^620^ Is Associated With Increased Frequency of Tregs in Blood

**Figure 7:**
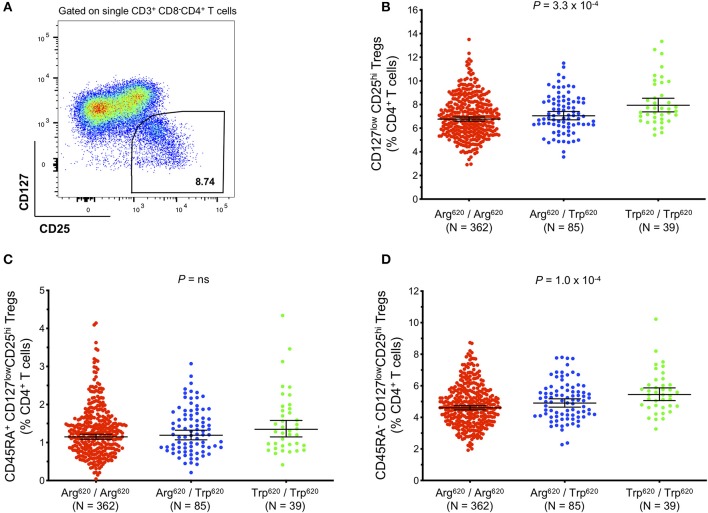
The autoimmunity missense PTPN22 Trp^620^ risk allele is associated with increased frequency of CD127^low^CD25^+^ Tregs in circulation. **(A)** Gating strategy depicting the delineation of total CD127^low^CD25^hi^ Tregs in blood. Data were obtained by surface immunostaining of freshly isolated whole blood from 486 healthy subjects selected by genotype from the Cambridge BioResource. **(B–D)** Scatter plots depict the frequency (geometric mean ± 95% CI) of: (i) CD127^low^CD25^hi^ Tregs **(B)**; (ii) CD45RA^−^ CD127^low^CD25^hi^ memory Tregs **(C)**; and (iii) CD45RA^+^ CD127^low^CD25^hi^ naive Tregs **(D)** in PTPN22 Arg^620^/Arg^620^ (*N* = 362; red), Arg^620^/Trp^620^ (*N* = 85; blue), and Trp^620^/Trp^620^ (*N* = 39; green) donors. *P*-values were calculated by linear regression analysis. ns, not significant.

The higher expression of *PTPN22* in CD8^+^ Teffs in SLE patients prone to flaring could be a mechanism to negatively regulate CD8^+^ Teff activation to reduce aggressive autoimmunity (17). To investigate whether the autoimmune-associated PTPN22 Trp^620^ risk allele could be altering T-cell activation potential and response to an autoimmune reaction in Tregs, we measured Treg frequency in fresh blood from our collection of 486 healthy donors from the recall-by-genotype Cambridge BioResource (CBR), where we were able to specifically obtain blood samples from rare (<2% in populations of European ancestry) Trp^620^/Trp^620^ homozygous individuals. Total Tregs were defined in this study according to the expression of the conventional Treg surface markers CD127 and CD25 (Figure 7A). We compared Treg frequencies in homozygous (*n* = 39) and heterozygous (*n* = 85) carriers of the missense Trp^620^ variant with homozygous carriers of the common *PTPN22* genotype encoding Arg^620^/Arg^620^ (*n* = 362). We found that the autoimmune PTPN22 Trp^620^ allele was significantly associated with an increased frequency of CD127^low^CD25^hi^ Tregs in blood (6.76, 7.05, and 7.94% in Arg^620^/Arg^620^, Arg^620^/Trp^620^, and Trp^620^/Trp^620^ donors, respectively; Figure 7B), with an estimated effect of 0.472% (Standard Error (SE) = 0.131; *P* = 3.3 × 10^−4^) increase in Treg frequency per copy of the minor Trp^620^ missense allele (Table 2). We noted that the frequency of Tregs in Arg^620^/Trp^620^ heterozygous donors was only marginally increased compared to the common Arg^620^/Arg^620^ donors, suggesting that two copies of the Trp^620^ missense allele may be necessary for the observed phenotype. Furthermore, we observed that the effect of the PTPN22 Trp^620^ allele was restricted to the CD45RA^−^ memory Treg subset (*P* = 1.0 × 10^−4^; Figure 7C), with no significant association being detected with the frequency of CD45RA^+^ naïve Tregs (Figure 7D).

**Table 2:** Genetic association of the PTPN22 Arg^620^Trp variant with Treg frequencies in blood.

**Cohort**	***N***	**MAF**	**Effect size**	**Standard error**	***P*-value**
Discovery (UK)	486	0.088^*^	0.472	0.131	3.3 × 10^−4^
Replication (Sardinia)	3,437	0.020	0.592	0.177	5.9 × 10^−4^
*Combined (Meta-analysis)^a^*	*3,923*	0.054^**^	*0.514*	*9.2 × 10^−3^*	*3.2 × 10^−6^*

*Genetic association of the PTPN22 Arg^620^Trp (rs2476601) with peripheral Treg frequencies*.

* *From 91 GBR individuals in 1,000 genomes phase III. Frequency is 0.164 in the 486 individuals from our discovery cohort with genotypes passing QC due to the targeted selection of rare homozygotes*.

** *Mean of the two population frequencies, not adjusting for sample size*.

a *Meta-analysis was performed using Fisher's method for combining P-values and inverse-variance weighting of parameter estimates. N, number of individuals with available PTPN22 Arg^620^Trp genotypes; MAF, minor allele frequency*.

To confirm this association we sought replication in a large independent population-based cohort from Sardinia (*n* = 3,437). Consistent with our findings, the PTPN22 Trp^620^ missense allele was significantly associated with increased Treg frequency in blood, with an estimated effect of 0.592% (SE = 0.177) per copy of the minor allele (*P* = 5.9 × 10^−4^; Table 2). The larger standard error in the replication set, in spite of much increased sample size, is due to the fact that Trp^620^/Trp^620^ homozygotes were not preferentially selected for as in the discovery set. The fairly large allele frequency difference between the Sardinian and UK populations may be due to differing levels of negative selection, with a greater selective disadvantage to the Trp^620^ risk allele in Sardinia. A meta-analysis of the combined datasets revealed an estimated increase of 0.514% in the frequency of Tregs in circulation per copy of the autoimmunity associated PTPN22 Trp^620^ allele (SE = 9.2 × 10^−3^, *P*_combined_ = 3.2 × 10^−6^; Table 2). Of note, analysis of the whole-blood eQTLGen database (34) revealed that the *PTPN22* Trp^620^ risk allele (rs247660C>T) was associated with the increased expression of a number of genes in different genomic loci (trans-eQTLs) in whole blood. Interestingly, among the top most significantly associated genes were several canonical Treg signature genes, such as *CTLA4, IL2RA*, and *FOXP3* (Supplementary Table 3). Gene ontology (GO) pathway analyses revealed an over 100-fold enrichment in terms associated with regulation of Treg differentiation, strongly suggesting that the effect of the *PTPN22* Trp^620^ missense allele on the expansion of Tregs is sufficient to be manifested in the expression of Treg genes in a highly heterogeneous whole-blood dataset. Moreover, we have recently reported that *PTPN22* is strongly expressed in human Treg subsets compared to memory Teff cells, and its expression was found to be strongly upregulated upon *in vitro* activation, particularly in activated Treg subsets (35). Consistently, we were able to detect increased *PTPN22* expression in total CD4^+^ T cells isolated from IFN^hi^ patients (Supplementary Figurse 6C,E), suggesting that it could reflect the expanded FOXP3^+^ Treg population in these patients.

To further validate that the increased frequency of total CD4^+^ Tregs observed in whole blood was due to the expansion of *bona-fide* thymically-derived HELIOS^+^FOXP3^+^ Tregs, we next performed intracellular immunophenotyping from cryopreserved PBMCs in a subset of healthy donors from the CBR, including 40 rare PTPN22 Trp^620^/Trp^620^ homozygotes, 39 Arg^620^/Trp^620^ heterozygotes and 73 Arg^620^/Arg^620^ common homozygotes, matched as closely as possible for age, sex and time of sample collection. In support of an expanded thymically-derived Treg population, we found that the frequency of HELIOS^+^FOXP3^+^ Tregs was increased in rare Trp^620^/Trp^620^ homozygous donors (4.32%) compared to common Arg^620^/Arg^620^ homozygotes (3.75%, *P* = 0.041; Supplementary Figure 7), a subset that we have previously shown to display a demethylated *FOXP3* TSDR (11). Similarly to HELIOS^+^FOXP3^+^ Tregs, we found an increased frequency of the less abundant HELIOS^−^FOXP3^+^ Tregs (*P* = 0.047; Supplementary Figure 7), suggesting a general increase in FOXP3^+^ memory Treg subsets in *PTPN22* Trp^620^/Trp^620^ individuals.

### Increased Expression of the Activation Marker PD-1 in Carriers of the PTPN22 Trp^620^ Missense Allele Is Associated With Reduced Treg Suppression Capacity and Increased T-Cell Proliferative Capacity

**Figure 8:**
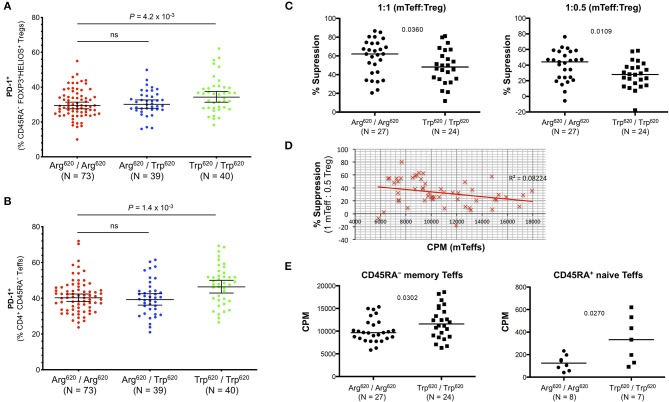
T cells from PTPN22 Trp^620^/Trp^620^ donors display an activated phenotype and increased proliferative capacity. **(A,B)** Scatter plots depict the frequency (geometric mean ± 95% CI) of PD-1^+^ cells within: (i) CD45RA^−^ CD25^hi^ FOXP3^+^HELIOS^+^ Tregs **(A)**; and (ii) CD45RA^−^ CD25^int/low^ Teffs **(B)** from 152 healthy volunteers recruited from the Cambridge BioResource. Data were stratified according to the genotype of the autoimmune-associated PTPN22 Arg^620^Trp variant. **(C)** Treg suppressive capacity was assessed in a subset of 27 common Arg^620^ homozygous and 24 age- and sex-matched rare Trp^620^ homozygous donors by measuring the proliferation of autologous CD45RA^−^ CD25^int/low^ T effector cells (mTeffs) in co-culture after *in vitro* activation for 6 days with anti-CD2/3/28 beads, at a ratio of either 1:1 or 1:0.5 mTeff:Treg **(D)** Increased proliferation of mTeffs, in the absence of Tregs, from a given donor correlates with reduced suppression of mTeff proliferation when autologous Tregs were present at a ratio of 1 mTeff: 0.5 Treg. **(E)** Proliferative capacity of CD45RA^−^ memory Teffs and CD45RA^+^ naïve Teffs was assessed after 6 days *in vitro* stimulation with anti-CD2/3/28 beads in the absence of Tregs in culture. *P*-values were calculated using two-tailed student's *t*-tests comparing the frequency of the assessed immune subsets between the PTPN22 Arg^620^/Arg^620^ and Trp^620^/Trp^620^ genotype groups. ns, not significant; mTeffs, CD45RA^−^ CD25^int/low^ T effector cells; CPM, proliferation readings—[^3^H]thymidine incorporation rate (fmoles/g).

In addition to the association with Treg frequency, we also found a significantly increased frequency of PD-1^+^ cells among the expanded CD45RA^−^FOXP3^+^HELIOS^+^ Tregs in the rare Trp^620^/Trp^620^ donors (34.3%) compared to common Arg^620^/Arg^620^ homozygotes (29.4%, *P* = 4.2 × 10^−3^; Figure 8A). Interestingly, we found a similar increase in the frequency of PD-1^+^ cells among the CD45RA^−^ Teffs in Trp^620^/Trp^620^ donors (*P* = 1.4 × 10^−3^; Figure 8B), suggesting that the PTPN22 Trp^620^ variant is associated with increased T-cell activation in both Tregs and Teffs. Furthermore, we noted that the expression of PD-1 was highly correlated between CD4^+^ CD45RA^−^ mTregs and mTeffs, and observed in all PTPN22 genotype groups. These results were observed both on the frequency of PD-1^+^ cells (Supplementary Figures 8A–C), but also quantitatively on the expression levels of PD-1 within PD-1^+^ cells (Supplementary Figures 8D–F). These data indicate that even in healthy controls, which display a noticeably lower frequency of PD-1^+^ cells compared to SLE patients, the expression of PD-1 on CD4^+^ CD45RA^−^ T cells is a sensitive marker of immune activation and putative ongoing inflammation.

To investigate if the increased expression of PD-1 in carriers of the missense Trp^620^ allele was functionally associated with their Treg suppression capacity *in vitro*, we sorted mTeffs (CD4^+^CD45RA^−^ CD127^+^CD25^int−low^) and autologous CD4^+^ CD127^low^CD25^hi^ Tregs from age- and sex-matched homozygous Trp^620^ and Arg^620^ donors, and stimulated them with α-CD2/3/28, before measuring mTeff proliferation after 6 days of culture. We observed an apparent reduction in the amount of suppression by Trp^620^ homozygous donors at two different ratios of mTeff:Tregs (1:1 or 1:0.5) cells, *P* = 0.0360 and *P* = 0.0109, respectively (Figure 8C).

These data lend further support to the hypothesis that Teff hyperactivity and increased proliferative capacity in carriers of the Trp^620^ variant renders them less amenable to suppression by the autologous Tregs. Consistently, increased Teff cell proliferation within a culture lacking Tregs correlated with less suppression when Tregs were present (Figure 8D). Furthermore, we found that Teff proliferation in Trp^620^ homozygous donors was slightly increased after *in vitro* T-cell stimulation (*P* = 0.0302; Figure 8E). Similarly, CD4^+^CD127^+^CD25^low^CD45RA^+^ naïve T cells from homozygous Trp^620^ donors also displayed increased proliferation compared to their Arg^620^ counterparts (*P* = 0.0270; Figure 8E). Taken together, these results suggest that Trp^620^ affects T-cell proliferation, therefore providing a potential mechanistic model supporting its genetic predisposition to autoimmunity.

## Discussion

The clinical heterogeneity associated with SLE has been a major challenge to our understanding of disease etiology and development of efficacious and long-lasting treatment options for patients with this debilitating condition. To address this issue, it is critical to develop better molecular diagnostic tools that would allow the advent of a personalized medicine approach in SLE. In this study, we have shed new light on the CD4^+^ FOXP3^+^ Treg compartment in SLE, and report an expansion of this population in patients. Our findings are in agreement with two previous publications reporting an expansion of thymically-derived memory FOXP3^+^HELIOS^+^ Tregs in patients, which was associated with increased disease activity (36, 37), and help elucidate the conflicting results that have been reported in the literature (8). Furthermore, we showed that FOXP3^+^ Tregs in patients display an activated phenotype marked by the increased frequency of PD-1^+^ cells, and their frequency was strongly correlated with the circulating concentrations of both a type I IFN marker, sSIGLEC-1, and IL-2. To our knowledge, this is the first time that an association between increased FOXP3^+^ Treg frequency and IL-2 concentrations is reported in SLE or in any other condition, in subjects not being treated with recombinant IL-2. The development of technologies with fg-level sensitivity (38) now provide the sensitivity to accurately measure physiological concentrations of IL-2, revealing important new insights into the regulation of IL-2 levels during the different stages of disease. Notably, with the exception of the IL-2 concentrations, which we were only able to measure in cohort 2, these results were observed in both a clinic-attending and a population-based cohort of patients not attending clinic and not in need of urgent medication, suggesting that this association is observed in both the active and remitting phases of the disease and represent a marker of disease activity.

Previously, we have hypothesized that a subset of non-conventional CD25^low^FOXP3^+^ Tregs, which are found to be increased in autoimmune patients, represents a terminal stage on a continuum of Treg differentiation marked by the gradual accumulation of immune activation/exhaustion markers such as PD-1 and a concomitant loss of Treg suppressive markers such as CTLA-4 and FOXP3 (11). In patients with active autoimmune disease, chronic immune activation promotes the accumulation of PD-1^+^ CD25^low^FOXP3^+^ Tregs as conventional activated FOXP3^+^HELIOS^+^ CD25^hi^ Tregs proliferate in an attempt to regulate autoreactive Teff hyperactivity. In this study we show that in addition to disease-specific mechanisms, the autoimmune-associated PTPN22 Trp^620^ missense allele also independently contributes to T-cell hyperactivity, marked by increased expression of PD-1 and increased Teff proliferative capacity. These data therefore support a Treg compensation model, whereby the combination of disease-specific and genetically-regulated predisposition for immune activation allow Teffs to be activated more readily and produce more IL-2, which would presumably promote Treg expansion. In SLE patients prone to flaring, increased *PTPN22* expression in activated CD8^+^ T cells is a biomarker of poor prognosis (17), which again indicates an activated phenotype in flaring autoimmunity. These data suggest that PTPN22 may also play an important regulatory role in maintaining transcriptional stability and silencing IL-2 signaling in activated Tregs. One hypothesis is therefore that the autoimmune-associated risk allele PTPN22 Trp^620^ could also directly contribute to the functional differentiation of Tregs, and the concomitant reduction in Treg suppressive capacity. Consistent with this hypothesis, we observed that the association between the PTPN22 Trp^620^ allele and increased Treg frequency was restricted to the memory Treg compartment, and not on the direct thymic output of naïve Tregs. However, we note that this study was conducted in an adult population with a median age of 55 years among rare PTPN22 Trp^620^/Trp^620^ donors, which may limit our capacity to assess the role of this variant on the generation of naïve CD45RA^+^ Tregs that are much more abundant in younger individuals. In addition, further work is required to test this functional mechanism and identify the direct role of Trp^620^ on Teff and/or Treg function (as well as other cell types) and their relative contribution to the observed increased risk of autoimmunity and observed Treg frequency.

One limitation of the current study is that, due to the lack of available surface-expressed markers, we were not able to isolate CD25^low^FOXP3^+^ Tregs from SLE patients to test their putative suppressive capacity *in vitro*. However, we note that the *in vitro* suppressive capacity of the heterogeneous CD127^low^CD25^low^ population has previously been demonstrated in humans, including in SLE patients (13, 39). Furthermore, in this study, we also show that the *FOXP3* TSDR of HELIOS^+^FOXP3^+^ CD127^low^CD25^low^ T cells is fully demethylated, which is consistent with their thymically-derived Treg origin. Nevertheless, given that even in SLE patients FOXP3^+^ cells represent at most 30–50% of the heterogeneous CD127^low^CD25^low^ T-cell population, it is critical to identify appropriate surface markers to unequivocally demonstrate the suppressive capacity of the different CD25^low^FOXP3^+^ T cell subsets, particularly within the HELIOS^−^ fraction.

In addition to T-cell intrinsic defects, the systemic type I IFN production could provide an additional mechanism to reduce Treg function in SLE patients. From an evolutionary perspective, it is important that type I IFN signaling can induce a temporary relief in the suppressive function of Tregs to promote an efficient anti-viral Teff response (40). The suppressive effect of IFN-α on Treg function has been shown to be mediated by the inhibition of cAMP signaling, without any further effect on their phenotype or TSDR demethylation profile (41). This mechanism has been proposed to contribute to the reduced Treg differentiation in SLE patients with an active type I IFN signature (42, 43), as well as to the increased incidence of secondary autoimmunity (44) and sustained reduction of Treg numbers (45) in patients treated with IFN-α. In contrast, we show that the frequency of FOXP3^+^ Tregs is strongly correlated with a type I IFN signature, as assessed by the concentration of the monocyte/macrophage activation biomarker sSIGLEC-1 (27). These data support a disease model whereby the synergistic effect of Teff hyperactivity and systemic IFN production impairs Treg function during flares of disease activity.

It has been previously reported that CD4^+^ Teffs isolated from SLE patients display a defect in IL-2 production when stimulated *in vitro*, through the transcriptional repression of the IL-2 gene (4, 46). Nevertheless, a longitudinal analysis of SLE patients has shown that this IL-2 production impairment could be relieved over the course of the disease—most notably in response to immunosuppressive treatments (47). Furthermore, it has been shown that the IL-2 secretion defect can be rescued by resting the CD4^+^ T cells from SLE patients prior to re-stimulation (48). Combined with our observation that circulating levels of IL-2 are strongly associated with increased FOXP3^+^ Treg frequencies, these data suggest that the homeostatic balance of IL-2 signaling is disrupted during disease flares in SLE patients. We therefore hypothesize that during disease flares, the pro-inflammatory changes promote increased CD4^+^ Teff function and IL-2 production and override the reported *in vitro* defect in IL-2 production by CD4^+^ Teffs. As a consequence, the expansion of the CD4^+^ FOXP3^+^ Tregs due to elevated IL-2 concentrations likely reflects an attempted compensatory mechanism to regulate Teff cell hyperactivity in an inflammatory environment during these periods of flaring autoimmunity. This is consistent with recent data from patients with active relapsing-remitting multiple sclerosis where Teff hyperactivity mediated by increased IL-6R signaling renders them resistant to Treg suppression (15). Together, these data support a therapeutic strategy in chronic relapsing autoimmune diseases, whereby temporary relief of Teff function during flares of active disease by anti-IL-6R or temporary T-cell immunoablation may be necessary to promote disease remittance and restore immune regulation, which could then be maintained through combination with low-dose IL-2 therapy.

Given the recent success of Treg-directed therapies, including low-dose IL-2 in SLE, and as more clinical trials move into phase 2 and 3 (49, 50), a key unanswered question is how Treg function and IL-2 homeostasis are restored. Currently, the studies that have been conducted in SLE targeted only patients with active disease and/or not responding to conventional standard-of-care treatment options. However, the increased IL-2 levels in SLE patients with active disease combined with our observation that Tregs may be refractory to further IL-2 stimulation (38), suggest that flaring SLE patients may not be the optimal target patient group to treat with exogenous IL-2. Instead, we hypothesize that low-dose IL-2 treatment could have maximal effect in well-managed patients, to maintain IL-2 homeostasis and prevent the recurrence of disease flares. Furthermore, the dosing regimen could also have profound effects in the efficacy of these treatment options. Current dosing strategies are primarily weekly treatment cycles of daily IL-2 treatment, adapted from the original study by Saadoun et al. (51) using low-dose IL-2 for the treatment of hepatitis C virus-induced vasculitis. We have shown previously that Tregs isolated from T1D patients treated with ultra-low doses of IL-2 are partially desensitized to further IL-2 stimulation after 24 h (38), suggesting that such a daily dosing schedule may not be the most efficient to promote a functional Treg response. We note that, He et al. administered IL-2 to patients every 2 days instead, which may have contributed to the increased efficacy reported in the study (24). Low-dose IL-2 studies and trials conducted to date in SLE were limited in patient numbers, and therefore it is not possible to accurately measure the specific impact of low-dose IL-2 or the non-specific concurrent immunosuppression on the management of the disease symptoms. Further trials in SLE and other autoimmune diseases specifically designed to investigate the effect of low-dose IL-2 on different stages of disease will be paramount to address these questions.

A better understanding of the functional heterogeneity of the Treg compartment and its modulation by IL-2 immunotherapy could also provide a mechanistic rationale to explore novel combination therapies to maximize the immunoregulatory potential of low-dose IL-2 therapy. One example could be the combination of IFN-α blockade, which is showing promise in the clinic (52, 53), in combination with low-dose IL-2 treatment. Patients with the highest levels of type I IFN signaling could benefit from short cycles of anti-IFN-α treatment during peaks of disease activity to prevent the chronic activation of the innate and adaptive immune response. Once in clinical remission, these patients could then benefit from low-dose IL-2 treatment instead, to maintain IL-2 homeostasis and Treg function, while minimizing the potential serious side-effects of blocking the IFN-α signaling pathway for long periods of time. Similarly, checkpoint agonism therapies such as PD-1 agonism could also be therapeutically beneficial in autoimmune diseases, and are under active investigation in early phase trials (54). Interestingly, PD-1 expression has been shown to be increased on Tregs in response to low-dose IL-2 treatment, and necessary for the maintenance of Treg regulatory homeostasis (55). Moreover, PD-1 signaling has been proposed to be central in maintaining Treg stability and the maintenance of a FOXP3 transcriptional profile (56). These data suggest that the combination of low-dose IL-2 immunotherapy with PD-1 agonism could be a promising treatment option to selectively enhance the suppressive capacity and regulatory homeostasis of the expanded Treg compartment, while suppressing the proliferation of the pathogenic autoreactive PD-1^+^ Teffs.

## Data Availability Statement

The datasets generated for this study are available on request to the corresponding author.

## Ethics Statement

The studies involving human participants were reviewed and approved by Peterborough and Fenland research ethics committee. The patients/participants provided their written informed consent to participate in this study.

## Author Contributions

RF, TT, LW, and JT designed experiments and interpreted data. RF, XC, JO, DR, JY, DT, MM, MS, VO, EF, DC, and MP performed experiments and analyzed the data. ST coordinated PTPN22 genotype-selected donor recruitment. FC and TV provided samples and clinical outcome data. RF, LW, and JT conceived the study and wrote the paper.

### Conflict of Interest

The authors declare that the research was conducted in the absence of any commercial or financial relationships that could be construed as a potential conflict of interest.
