# Supplementary material for: Chronic Immune Activation in Systemic Lupus Erythematosus and the Autoimmune PTPN22 Trp620 Risk Allele Drive the Expansion of FOXP3+ Regulatory T Cells and PD-1 Expression
Source: Front Immunol. 2019 Nov 8;10:2606. doi: 10.3389/fimmu.2019.02606 (PMC6857542; doi:10.3389/fimmu.2019.02606)
Supplement: Supplementary file 1 [file Data_Sheet_1.docx]

***Supplementary material***

**Supplementary Table 1. Antibodies and immunostaining panels used for flow cytometry.** Detailed description of the fluorochrome-conjugated and oligo-tagged antibodies used for both immunostaining panels and single-cell RNA-sequencing in this study.

**Supplementary Table 2. Transcriptional profile of ex vivo isolated CD4+ T cells following stimulation with PMA + ionomycin.** Normalised counts of the 594 markers present in the NanoString Human Immune panel. Data was generated from total CD4+ T cells isolated ex vivo from four selected SLE patients with high type I interferon transcriptional signature (IFN^hi^), two SLE patients with low IFN signature (IFN^low^) and four healthy donors, following in vitro stimulation with PMA + ionomycin.

[See attached excel file for complete dataset]

**Supplementary Table 3. Top 10 PTPN22 rs2476601 trans-eQTLs in whole blood.** Top 10 trans-eQTLs in whole blood for the *PTPN22* Arg620Trp (rs2476601) missense allele. Data was extracted from the eQTLGen database (ref. 28) measuring the expression of 19,960 genes in whole blood and the correlation with 10,317 trait-associated SNPs.

**Supplementary Figure 1. FOXP3^+^HELIOS^+^ Tregs from SLE patients in both the CD25^low^ and CD25^hi^ compartments display a demethylated FOXP3 TSDR.**  **(A)** Gating strategy for the FACS sorting of CD45RA^−^ memory CD25^low^ (red) and CD25^hi^ (blue) FOXP3^+^HELIOS^+^ Tregs. **(B)** Frequency (mean +/− SEM) of reads demethylated at eight or nine of the nine interrogated CpG sites in the *FOXP3* TSDR. The data were obtained from sorted cells from three independent female SLE patients recalled from the Cambridge BioResource. Note that because all data was obtained from female donors, X-chromosome inactivation causes half of the reads to be methylated and a correction factor of two was applied to obtain the frequency of demethylated reads. We also observed an unexpected high rate of FOXP3 TSDR demethylation on sorted CD45RA^−^ Teffs in one of the patients (4.7% after adjustment for X-chromosome inactivation). This was due to the fact that we did not exclude the rare FOXP3^+^HELIOS^+^ cells from the sorted CD45RA^−^ Teff population, which are known to be demethylated at the TSDR and was found at an unusually high frequency in the SLE patient with residual TSDR demethylation.

**Supplementary Figure 2. Characterising the heterogeneity of human CD4^+^ CD127^low^CD25^low^ T cells at the single-cell level.**  **(A)** Heatmap displaying the top 10 differentially expressed genes in each identified CD4^+^ T-cell cluster. Data was extracted from a targeted single-cell RNA-sequencing dataset (ref. 29), and depicts the analysis of pre-sorted CD127^low^CD25^hi^ (N = 7,711) and CD127^low^CD25^low^ (N = 7,115) T cells from one SLE patient and two control donors, including one type 1 diabetes (T1D) patient and one healthy donor. **(B,C)** Frequency of FOXP3^+^ cells (as assessed by the expression of >= 1 copy of *FOXP3* at the mRNA level) from either the CD127^low^CD25^hi^ conventional Treg gate (**B**) or the CD127^low^CD25^low^ population (**C**) in each identified T-cell cluster. Tregs; regulatory T cell clusters; Teff, effector T-cell clusters; SLE, systemic lupus erythematosus; T1D, type 1 diabetes; HC, healthy control

**Supplementary Figure 3. Expression of the activation marker PD-1 is highly correlated in the CD45RA^−^ memory Treg and Teff populations.** (**A,B**). Data shown depicts the correlation between the frequency of PD-1^+^ cells in CD45RA^−^ CD25^hi^ FOXP3^+^HELIOS^+^ Tregs and CD45RA^−^ Teffs in both SLE patients (**A**) and healthy controls (**B**). The correlation coefficients (r) and the respective *P* values from the linear regression are shown in the plots. (**C, D**) Scatter plots (geometric mean +/- 95% CI) depict the PD-1 mean fluorescence intensity (MFI) in the PD-1^+^ cells within CD45RA^−^ CD25^hi^ FOXP3^+^HELIOS^+^ Tregs (mTregs; depicted in red) and CD45RA^−^ Teffs (mTeffs; depicted in black). Data was obtained from the intracellular immunostaining of 34 SLE patients and 24 healthy volunteers from the clinic-attending discovery cohort 1 (**C**) and 41 SLE patients and 112 healthy volunteers from the population-based replication cohort 2 (**D**). *P* values were calculated using paired two-tailed student’s t-tests comparing the PD-1 MFI within the respective mTreg and the mTeff subsets. SLE, systemic lupus erythematosus; HC, healthy control; ns, not significant.

**Supplementary Figure 4. Immunophenotyping CD45RA^−^ FOXP3+HELIOS+ Tregs in SLE patients.** (**A,B**) Scatter plots depict the frequency (geometric mean +/- 95% CI) of CD15s^+^ (**A**) and CD226^+^ (**B**) cells within the expanded subset of CD25^hi^ FOXP3^+^HELIOS^+^ CD45RA^−^ memory Tregs from 34 SLE patients (red) and 24 healthy volunteers (blue) from the clinic-attending discovery cohort 1. (**C, D**) Scatter plots depict the frequency (geometric mean +/- 95% CI) of CD161^+^ (**C**) and HLA-DR^+^ (**D**) cells within the expanded subset of CD45RA^−^ CD25^hi^ FOXP3^+^HELIOS^+^ Tregs from 41 SLE patients (red) and 112 healthy volunteers (blue) from the population-based replication cohort 2. *P* values were calculated using two-tailed student’s t-tests comparing the frequency of the assessed immune subsets in patients and controls. SLE, systemic lupus erythematosus; HC, healthy control; ns, not significant.

**Supplementary Figure 5. Memory CD4^+^ CD45RA^−^ T effector (Teff) cells from SLE patients display increased expression of immune activation markers.** (**A-D**) Scatter plots depict the frequency (geometric mean +/- 95% CI) of: (i) PD-1^+^ (**A**); (ii) ki-67^+^ (**B**); (iii) TIGIT^+^ (**C**); and (iv) CD15s^+^ (**D**) cells within CD45RA^−^ CD4^+^CD25^int/low^ Teffs. Data was obtained from a discovery clinic-attending cohort (cohort 1) consisting of 34 SLE patients (depicted in red) and 24 healthy volunteers (depicted in blue) and from a population-based replication cohort (cohort 2) consisting of 41 SLE patients and 112 healthy volunteers. *P* values were calculated using two-tailed student’s t-tests comparing the frequency of the assessed immune subsets in patients and controls. SLE, systemic lupus erythematosus; HC, healthy control; ns, not significant.

**Supplementary Figure 6. Regulatory T cell (Treg) expansion and type I interferon (IFN) signature can be detected at the transcriptional level in *ex vivo* isolated CD4^+^ T cells.** (**A**) Scatter plots depict the distribution (median) of the type I interferon (IFN) transcriptional signature (as previously measured in PBMC – ref. 27) and frequency of FOXP3+ cells within either CD4^+^ T cells or CD127^low^CD25^low^ T cells from a subset of: (i) four SLE patients with a high transcriptional IFN signature (IFN^hi^; depicted in red); (ii) two SLE patients with low IFN signature (IFN^low^; depicted in blue); and (iii) four healthy donors (depicted in black). (**B-D**). Correlation between the detected transcriptional expression of canonical IFN signature genes (**B**), regulatory T cell (Treg) signature genes (**C**) and T-cell activation genes (**D**) and the assessed quantitative transcriptional IFN signature. Transcriptional data was obtained from total CD4^+^ T cells following *in vitro* stimulation with PMA + ionomycin using the NanoString platform. The correlation coefficients (r) and the respective *P* values from the linear regression are shown in the plots. (**E**) Scatter plots depict the distribution (median) of the assessed Treg signature genes in the IFN^hi^, IFN^lo^ and HC groups. (**F,G**) Data shown depicts the correlation between the assessed Treg signature genes and the frequency of FOXP3^+^ cells assessed at the protein level by flow cytometry in either total CD4^+^ T cells (**F**) or CD4^+^ CD127^low^CD25^low^ T cells (**G**). AU, arbitrary units; SLE, systemic lupus erythematosus; HC, healthy control; IFN, type I interferon; ns, not significant.

**Supplementary Figure 7. The PTPN22 Trp^620^ missense risk allele is associated with an increased frequency of thymically-derived Tregs with a fully demethylated FOXP3 TSDR.** Data shown depict the gating strategy and distribution (geometric mean +/- 95% CI) of: (i) FOXP3^+^HELIOS^+^; and (ii) FOXP3^+^HELIOS^−^ CD45RA^−^ CD4^+^CD127^low^CD25^hi^ Tregs. Data was generated by intracellular immunostaining of cryopreserved PBMCs from a subset of 73 Arg^620^/Arg^620^ (red), 39 Arg^620^/Trp^620^ (blue) and 40 Trp^620^/Trp^620^ (green) healthy donors recruited from the Cambridge BioResource, and was stratified according to the genotype at the autoimmune-associated PTPN22 Arg^620^Trp variant. *P* values were calculated using two-tailed student’s t-tests comparing the frequency of the two Treg subsets between the PTPN22 Arg^620^/Arg^620^ and Trp^620^/Trp^620^ genotype groups.

**Supplementary Figure 8. PD-1 expression on CD4^+^ T cells is a biomarker of immune activation.** (**A-C**) Data shown depicts the correlation between the frequency of PD-1^+^ cells within CD45RA^−^ CD25^hi^ FOXP3^+^HELIOS^+^ Tregs (mTregs) and CD45RA^−^ Teffs (mTeffs) in: (i) 73 Arg^620^/Arg^620^ (**A**); (ii) 39 Arg^620^/Trp^620^ (**B**); and (iii) 40 Trp^620^/Trp^620^ (**C**) healthy donors recruited from the Cambridge BioResource. (D-F) Data shown depicts the correlation between the PD-1 mean fluorescence intensity (MFI) in the PD-1^+^ cells within CD45RA^−^ CD25^hi^ FOXP3^+^HELIOS^+^ Tregs (mTregs) and CD45RA^−^ Teffs (mTeffs) in: (i) 73 Arg^620^/Arg^620^ (**D**); (ii) 39 Arg^620^/Trp^620^ (**E**); and (iii) 40 Trp^620^/Trp^620^ (**F**) healthy donors recruited from the Cambridge BioResource. The correlation coefficients (*r*) and the respective *P* values from the linear regression are shown in the plots.
